# Harnessing the potential of lipid nanoparticles for the delivery of chemically modified siRNA to combat hepatic adenovirus infection

**DOI:** 10.1016/j.omtn.2023.06.005

**Published:** 2023-06-28

**Authors:** Quazi T.H. Shubhra, A.K.M. Moshiul Alam

**Affiliations:** 1 Institute of Chemistry, University of Silesia in Katowice, 41-500 Chorzów, Poland; 2 Institute of Radiation and Polymer Technology, Bangladesh Atomic Energy Commission, Dhaka 1000, Bangladesh

**Keywords:** lipid nanoparticles, hepatic infection, siRNA, drug delivery system, human adenovirus

Lipid nanoparticles (LNPs) represent a highly versatile class of drug delivery system (DDS) for both small-molecule drugs and nucleic acids, thus presenting a promising avenue for targeted therapeutic interventions. A recent study, conducted by Geisler et al. and published in the journal *Molecular Therapy – Nucleic Acids*, sheds light on the therapeutic potential of LNPs as carriers for the delivery of anti-adenoviral small interfering RNA (siRNA) in the context of hepatic human adenovirus (hAd) serotype 5 (hAd5) infection.^1^ The findings of this investigation demonstrate a compelling strategy for combating hepatic hAd5 infection by utilizing effective 2′-O-methyl siRNA modifications and its encapsulation within LNPs, offering promising potential in treating this debilitating disease.

The available treatment options for hepatic hAd infections are currently limited, lacking FDA- or EMA-approved antiviral therapies. Off-label usage of drugs such as brincidofovir (BCV), ribavirin, cidofovir (CDV), and ganciclovir only provides limited benefits, with CDV and BCV being associated with nephrotoxicity and gastrointestinal toxicity,^2^ respectively. Although personalized hAd-specific T cell therapy exhibits potential, its practical implementation is impeded by cost and time constraints. Therefore, there is an urgent need for the development of innovative therapies, and it is worth exploring promising DDSs such as liposomes, polymeric nanoparticles, dendrimers, and conjugates^3^ for potential effectiveness in treating hepatic hAd infections. LNPs present a promising solution as a versatile DDS for RNA interference-based antiviral therapy. Properly designed LNP-based DDSs can hold strong potential in the treatment of hepatic hAd5 infection, offering numerous advantages, e.g., biocompatibility. LNPs exhibit the capability to safeguard the enclosed drug/nucleic acid against degradation. Moreover, LNP formulations can be engineered to enable specific cellular uptake, allowing for precise delivery to desired tissues or cell types.

In their study, Geisler et al. conducted a meticulous investigation to develop properly designed LNPs for the treatment of hepatic hAd5 infection. Through *in vitro* experiments, they demonstrated the effective inhibition of adenoviral replication (95%–98%) using specific siRNAs targeting adenoviral genes, namely pTP and Pol (siPol-1, siPol-2, and sipTP; where pTP represents pre-terminal protein and Pol represents adenoviral proteins DNA polymerase). A key objective of the study was to identify siRNAs capable of targeting multiple hAd subtypes, and after extensive analysis of various subgroups, serotypes, and adenoviral target sequences, sipTP emerged as a promising candidate. To overcome the challenge of *in vivo* siRNA delivery, LNPs were employed (LNP-sipTP_mod_), exhibiting high encapsulation (90%) and an appropriate size (<90 nm). The incorporation of PEG (polyethylene glycol) lipids in their developed LNPs is noteworthy, as PEG is widely recognized for its ability to greatly reduce clearance mediated by reticuloendothelial cells.^4^

**Figure 1:**
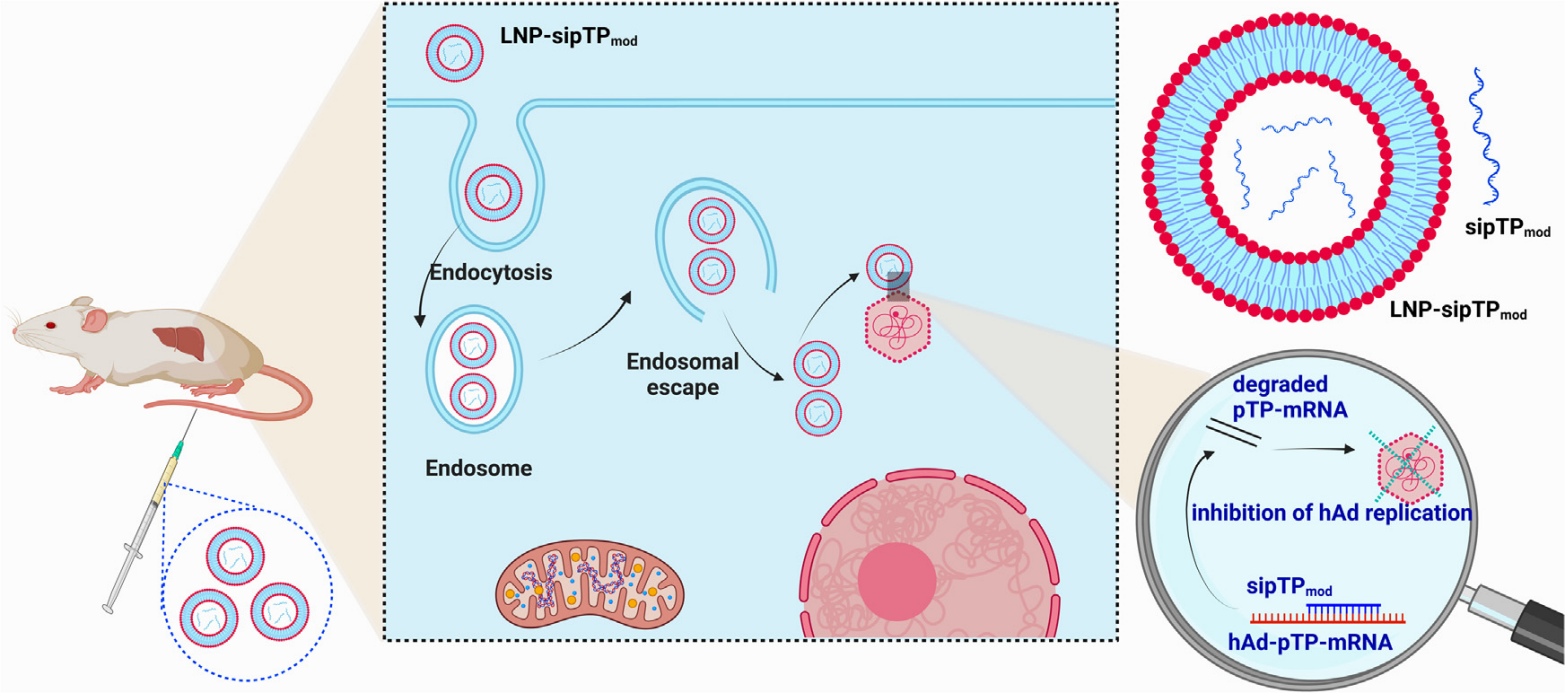
Schematic illustration demonstrating the potent inhibition of virus replication through LNP-sipTP_mod_ Upon uptake by hepatocytes, sipTP_mod_ effectively silences hAd-pTP-mRNA, suppressing hAd replication to mitigate hepatic hAd5 infection.

LNP-sipTP_mod_ showed promise for combating hepatic hAd5 infection when investigated in an immunosuppressed Syrian hamster model following a mechanism shown in Figure 1. A single intravenous administration of LNP-sipTP_mod_ effectively inhibited hepatic hAd5 infection, leading to reduced viral titers in the liver and serum compared with controls. LNP-sipTP_mod_ also exhibited a trend toward decreased liver inflammation along with reduced liver injury. The results demonstrated few intriguing findings.

(1) In a low dose infection model, LNP-sipTP_mod_ demonstrated reduced weight loss (up to 7.6%) and significantly decreased the virus levels compared with LNP-siContr_mod_ controls (up to 13.7% weight loss). The liver tissue of LNP-sipTP_mod_-treated hamsters exhibited lower pathological scores. Furthermore, hAd5 titers in the serum and liver were 9.4- and 3.4-fold lower, respectively, in the LNP-sipTP_mod_-treated group compared with the LNP-siContr_mod_-treated group.
(2) In a moderate dose infection model (10-fold higher than the low dose), LNP-sipTP_mod_ showed reduced weight loss and significantly decreased viral titers in the liver, serum, and spleen, as confirmed by immunohistochemical staining. Compared with the low dose model, there was a remarkable reduction of 19.6-fold, 100-fold, and 110-fold in viral titers observed in the liver, spleen, and serum, respectively.
(3) In comparison to AAV-amiR 348 (adeno-associated virus artificial microRNA 348) treatment, LNP-sipTP_mod_ exhibited higher efficacy in reducing hAd5 titers, potentially attributed to LNPs' enhanced liver targeting and direct cytoplasmic entry of siRNAs. AAV-amiR 348 achieved a 50% reduction, whereas LNP-sipTP_mod_ achieved a greater reduction of 70.5%.

siRNA therapeutics, based on Nobel Prize-winning science, enable sequence-specific downregulation of target gene expression through targeted mRNA degradation.^5^ However, their clinical application faces significant challenges due to rapid enzymatic degradation of RNA, primarily catalyzed by ribonucleases.^6^ RNA instability arises from the susceptibility of ribose sugar’s 2′-OH group to base-catalyzed hydrolysis, leading to phosphodiester bond degradation. To address this, Geisler et al. introduced modifications to siRNAs, incorporating 2ʹ-O-methyl residues and phosphorothioate-linkages at specific positions. These modifications, yielding siPol_mod_ and sipTP_mod_, not only enhance stability against endonucleases but also prevent immune responses, thereby improving siRNA performance.

In addition to LNP formulations, other platforms, such as calcium phosphate (CaP) NPs and N-acetylgalactosamine (GalNAc)-ligand-modified siRNAs, have emerged as widely utilized strategies. Infusion fluids that can be directly injected into the human body were employed by us to prepare CaP NPs for efficient delivery of nucleic acid.^7^ GalNAc-conjugated siRNA utilizes ligand-mediated targeting of the asialoglycoprotein receptor, which is highly expressed on hepatocyte surfaces, facilitating extensive uptake by hepatocytes.^8^ Compared with LNPs, GalNAc conjugates offer the advantages of being relatively small and well-defined in structure.

Undoubtedly, modified siRNAs with enhanced stability represent a highly favored approach for the treatment of liver diseases. The work by Geisler and co-workers serves as a prime example, demonstrating the effective treatment of hepatic hAd5 infection through the development of LNPs for the delivery of modified siRNAs. Future investigations in this field could explore the utilization of multifunctional NPs,^9^ which hold great promise for advancing preclinical and clinical applications. Multifunctional NPs possess the capability to integrate multiple functionalities within a single NP, offering distinct advantages over conventional NPs, particularly in the context of image-guided therapy.^10^ By co-entrapping imaging agents with siRNAs, this approach could enable both therapy guidance and the tracking of biodistribution. Notably, the clinical translation of such a strategy would likely be more cost-effective compared with alternative approaches like T cell therapy. Therefore, further exploration of this avenue is warranted to bridge the gap between benchside research and bedside applications.
